# Evaluating the Effect of Rail Fastener Failure on Dynamic Responses of Train-Ballasted Track-Subgrade Coupling System for Smart Track Condition Assessment

**DOI:** 10.3390/ma15072675

**Published:** 2022-04-05

**Authors:** Yuanjie Xiao, Zhenxing Chang, Jianfeng Mao, Sijia Zhou, Xiaoming Wang, Weidong Wang, Degou Cai, Hongwei Zhu, Yao Long

**Affiliations:** 1 School of Civil Engineering, Central South University, Changsha 410075, China; yjxiao@csu.edu.cn (Y.X.); czxfst@csu.edu.cn (Z.C.); zhousj@csu.edu.cn (S.Z.); xiaoming-wang@csu.edu.cn (X.W.); 147745@163.com (W.W.); 2 Ministry of Education Key Laboratory of Engineering Structures of Heavy Haul Railway, Central South University, Changsha 410075, China; 3 Railway Engineering Research Institute, China Academy of Railway Sciences Corporation Limited, Beijing 100081, China; zhuhongwei@yeah.net; 4 School of Railway Engineering, Hunan Technical College of Railway High-Speed, Hengyang 421002, China

**Keywords:** high-speed railway, rail fastener failure, train-ballasted track-subgrade coupling system

## Abstract

Rail fasteners are among the key components of ballasted track of high-speed railway due to their functionality of fixing rails to sleepers. The failure of rail fastening system hinders the transmission of train loads to underlying track substructure and therefore endangers the operation safety and longevity of ballasted track. This paper first established a three-dimensional (3D) numerical model of the train-ballasted track-subgrade coupling system by integrating multibody dynamics (MBD) and finite element method (FEM). Numerical simulations were then performed to investigate the effects of different patterns of rail fastener failure (i.e., consecutive single-side, alternate single-side, and consecutive double-side) on critical dynamic responses of track structures, train running stability, and operation safety. The results show that the resulting influences of different patterns of rail fastener failure descend in the order of consecutive double-side failure, consecutive single-side failure, and alternate single-side failure. As the number of failed fasteners increases, the range where dynamic responses of track structures are influenced extends, and the failure of two consecutive single-side fasteners exerts a similar influence as that of four alternate single-side fasteners. The failure of single-side fasteners affects dynamic responses of the intact side of track structures relatively insignificantly. The influence of rail fastener failure on track structures exhibits hysteresis, thus indicating that special attention needs to be paid to locations behind failed fasteners during track inspection and maintenance. The occurrence of the failure of two or more consecutive fasteners demands timely maintenance work in order to prevent aggravated deterioration of track structures. The findings of this study could provide useful reference and guidance to smart track condition assessment and condition-based track maintenance.

## 1. Introduction

Running stability and safety of railway vehicles are the primary goals of railroad track inspection and maintenance, especially for high-speed railroad. The existence of commonly observed track defects, including failed fasteners, unsupported sleepers, and differential track settlement needs to be identified in a timely manner and rectified properly in order to prevent significant casualties, economic losses, and social impacts. The rail fastening system is one of the key components of railroad track structures. It fixes rail beams to sleepers (or cross-ties), which facilitates not only restricting longitudinal, transverse, and vertical movement and vibration of railway track, but also transmitting train loads to underlying track substructures evenly. The additional key function of rail fasteners in the railway track system is to reduce the noise related to high-speed train and track structures. Wang et al. [1] analyzed the vibration reduction effect of a suspension fastener in subway track in both time and frequency domains and found that the use of this new type of fastener can significantly reduce track vibration. The functionality and durability of rail fasteners are affected by factors including fastener stiffness, the clips of the fastening system, and temperature. Li et al. [2] considered the wheel–rail interaction by focusing on the relative differences between the two fastener systems only and comparing the experimental and numerical results. They found that the fastener stiffness significantly affects the indoor environment noise of railway vehicles, i.e., the lower the fastener stiffness is, the greater the indoor environmental noise is. The temperature could also influence the characteristics of the rail fastening system. Wang et al. [3] studied the influences of ambient temperature on rail fasteners and dynamic responses of railway track in the frequency domain. Liu et al. [4] treated track irregularity as fixed-point excitation to study the temperature sensitivity of rubber pad with low-resistance WJ-7B fasteners used in high-speed railway tracks. Their results showed that the stiffness of rail fasteners was sensitive to low temperature, remained stable at high temperature, and showed little impact on the vertical vibration of high-speed railway track. However, the majority of existing studies are based on two-dimensional (2D) train-track coupling model only, which is still different from the reality. Liu et al. [5] considered the nonlinearity of under-sleeper pads (USP) used in high-speed railway tracks, studied the influence of USP parameters on dynamic responses of the vertical train-track–viaduct coupling system, and then analyzed the temperature effect of USP. Yuan et al. [6] established a random dynamic model of heavy-haul train running in small-radius curve section to study the durability of rail fasteners. Their results show that the rail fastening system studied is basically durable and reliable with part of them possessing the risk of pulling screws out.

The rail fastener failures were previously studied in the literature with a focus on the stiffness and damping properties of rail fasteners, which indeed are particularly important for high-speed railway. Wei et al. [7] studied the vibration law affected by the USP stiffness of the rail fastening system by using the vertical train-track coupling model. Shi et al. [8] realized the coupling between train and track by establishing a double-layer track model and then studied the influence of USP stiffness on dynamic responses of heavy-haul railway track. They concluded that the service life of heavy-haul railway track can be prolonged by increasing the USP stiffness, which may also be applicable for high-speed railway track. By studying the stiffness and damping properties of rail fasteners, Kun et al. [9] concluded that increasing the fastener stiffness can reduce, to a certain extent, the vibration of the bridge structure in the train–bridge coupling system but increase the rail vibration on the other hand. However, the damping effect of increasing fastener damping is relatively weak. Note that the input of train loading used in their study was just in the form of external excitation, which is still different from the real coupling interaction between railway vehicles and bridge. Gao et al. [10] built the first high-frequency, dynamic-stiffness testing system for elastic elements of the rail fastening system, and obtained the vertical and horizontal stiffness and loss coefficients (which are related to dynamic frequency) of high-speed railway W300 fasteners at 5–1250 Hz. This study provides the possibility of conducting refined analysis of rail fasteners. Fermér et al. [11] studied the interaction between train and track with soft and stiff rail pads by carrying out full-scale experiments, from which it was concluded that the stiffness of rail pads could greatly affect dynamic contact forces; however, this study focused on vertical direction only. Germonpré et al. [12] compared the influence of longitudinal track unevenness and track stiffness variation on railway vibration and concluded that track stiffness variation can be modelled as equivalent track unevenness when they occurred simultaneously. When studying degradation of railway track geometry, Nielsen et al. [13] analyzed the relationship between track stiffness gradient and differential settlement, then proposed a method for measuring track vertical stiffness in longitudinal direction, which can efficiently minimize the life cycle cost and environmental footprint of railway track maintenance. Even though they analyzed the stiffness of rail pads, their study only focused on vertical direction and did not recommend any maintenance measures. Ngamkhanong and Goto [14] compared the functionalities of new and aged rail pads and concluded that the deterioration of rail pads appeared to significantly increase rail/sleeper contact forces rather than wheel/rail contact forces, and that softer pads exhibited better performance in terms of reducing dynamic responses.

The loosened or failed rail fasteners commonly exist throughout the entire railway service life. A significant number of studies have been dedicated to understanding external and internal mechanisms of rail fastener failures and potential detrimental consequences to railway operation safety. Gao et al. [15] and Liu et al. [16] reported the internal mechanical mechanisms of rail fastener failure so as to provide technical countermeasures for high-speed railway operation and maintenance in the presence of rail fastener failure. Xiao et al. [17] modeled the railway track as discretely supported asymmetric elastic beams with stiffness varying along the track and modeled the half vehicle as a multi-body system, where the reduction coefficients for stiffness and damping properties of rail fasteners were also introduced. They obtained dynamic track responses such as displacement, velocity, acceleration and wheel/rail contact force from numerical simulations and then calculated the derailment coefficient and changing rate of wheel tread contact point. However, the interaction between rail beams and sleeper modeled by them failed to consider the longitudinal restraining effect of rail fasteners on railway track. Remennikov and Kaewunruen [18] investigated the influence of another key component of the rail fastening system, namely the rail pads, while other components of the rail fastening system such as fasteners and clips were not included in their scope. Kaewunruen et al. [19] categorized different types of failures of the rail fastening system and proposed a risk-based approach driven by a risk management framework; however, extending this approach to other failure modes still requires extra caution. Xu et al. [20] studied the influence of rail fastener failure on wheel–rail interaction and dynamic track responses with the influence of random track irregularity considered similarly. Their calculation results show that when the number of failed rail fasteners exceeded three, the wheel–rail interaction and track vibration were greatly impacted. Morales-Ivorra et al. [21] calibrated rail fastener stiffness from field testing results, simulated the dynamic impact of rail fastener failure on wheel–rail contact by using the commercial software program Vampire^®^, and evaluated the risk of train derailment on curved railway track. Their simulation results show that rail fastener failure has little impact on train running stability. With reference to three particular defect sites on a ballasted railway track in the U.K., Thompson et al. [22] proposed maintenance and repair strategies at such locations, e.g., the deflections measured during the train passages were used to determine the thickness of shims placed between the rail pad and the sleeper. Zhang et al. [23] analyzed the effects of fastener looseness and failure from energy perspective, calculated acceleration responses by setting different levels of fastener looseness, and then proposed the algorithm for identifying rail fastener looseness from Hilbert-Huang Transform (HHT) energy entropy. Note that they analyzed only the vertical acceleration, while the analyses of longitudinal and transverse acceleration responses still remain unexplored.

From the above-mentioned survey of existing literature on rail fastener failure, it can be seen that the inherent mechanical mechanism of rail fastener failure was previously studied from FEM simulations [21,23], mainly because it is very challenging to study it from laboratory or field tests. Further, such previous numerical simulations focused on the vertical train-track coupling only with the longitudinal motion of wheel-sets rarely considered, and few longitudinal vibration analyses of the train-ballasted track coupling system existed in the literature. The vibration analysis of the 3D train-ballasted track-subgrade coupling system is barely studied, while the majority of the related studies focused on vibration analysis of railway track on bridges rather than on that of railway track geo-foundation layers (e.g., embankment and subgrade soils). By establishing an innovative numerical model of the 3D train-ballasted track-subgrade coupling system, this paper studies the influence of rail fastener failure on dynamic track responses and assesses detrimental consequences of rail fastener failure to the operation safety of railway vehicles and the stability of track structures accordingly.

The remaining sections of this paper are organized as follows. In Section 2, the establishment of the 3D numerical model of the train-ballasted track-subgrade coupling system is described in details. Section 3 compares the field test data against model simulation results to verify the established numerical model of the coupling system. Section 4 further discusses the influence of different fastener failure patterns on dynamic responses of the coupling system. Finally, Section 5 summarizes the major findings and conclusions of this study.

## 2. Numerical Modelling of the 3D Train-Ballasted Track-Subgrade Coupling System

### 2.1. Hypothesis of the Model Developed

- The train ran on the track at a constant speed. The wheelsets retained full contact with the rail surface and there existed no sliding, climbing derailment, or jumping derailment.
- The vehicle bodies, bogies, and wheel-sets were assumed as rigid bodies with the eccentricity of vehicle gravity ignored. The stiffness and damping properties of the vehicle springs between the first and second suspensions were assumed linear.
- The rail was modeled by spatial Euler–Bernoulli beam elements of the finite element method (FEM), and 6 degrees of freedom were considered for each node.
- Only one representative track irregularity sample was selected for use as wheel-rail excitation.
- Sleepers and ballast layers were assumed to be mass blocks. In the lateral and vertical directions, linear spring-dashpots were used to connect the rail and sleepers, sleepers and ballast layer, ballast layer and subbase, as well as subbase and subgrade, respectively. The shear forces transmitted within ballast layers were also modeled by linear spring-dashpots.

### 2.2. High-Speed Train Model

**Figure 1:**
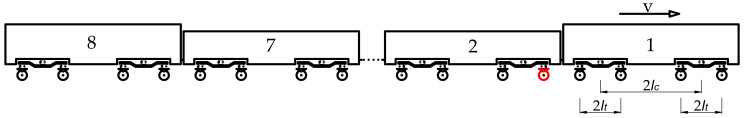
Illustration of the configuration of high-speed trains modeled in this study.

**Figure 2:**
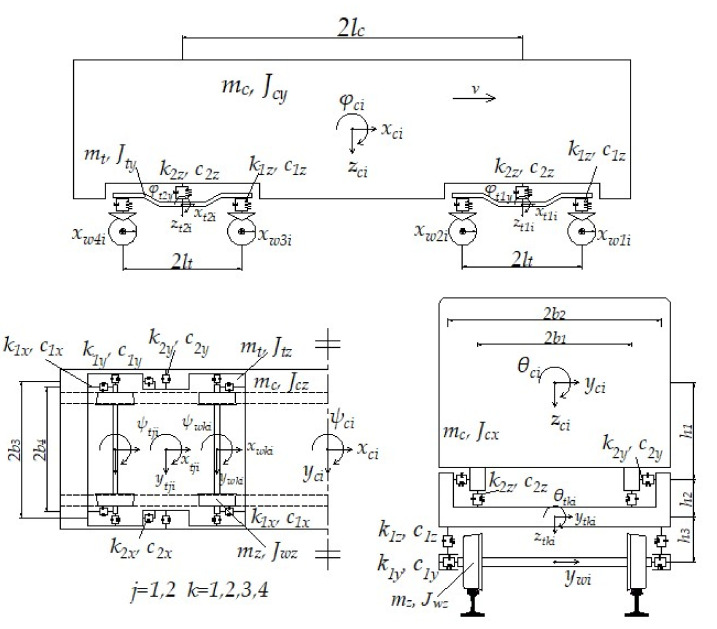
Illustration of the 3D high-speed railway vehicle model developed in this study.

The high-speed train modeled in this study consisted of two locomotives (No. 1 and 8) and six passenger cars (No. 2 to 6), whose configuration is sketched in Figure 1. Each of the railway vehicles consisted of one car body, two bogies, four wheel-sets, and the spring-dashpot connections between the first and second suspensions [24], as shown in Figure 2. Note that all the symbols included in Figure 2 were defined in Appendix A, along with other essential model parameters. Each car body or bogie had six degrees of freedom (DOFs), which were denoted by the longitudinal displacements $x_{ci}$ and $x_{t_{j}i}$, lateral displacements $y_{ci}$ and $y_{t_{j}i}$, vertical displacements $z_{ci}$ and $z_{t_{j}i}$, roll displacements $\theta_{ci}$ and $\theta_{t_{j}i}$, pitch displacements $\varphi_{ci}$ and $\varphi_{t_{j}i}$, and yaw displacements $\psi_{ci}$ and $\psi_{t_{j}i}$, respectively. Each wheel-set had three DOFs, i.e., longitudinal displacement $x_{w_{k}i}$, lateral displacement $y_{w_{k}i}$, and yaw displacement $\psi_{w_{k}i}$ (where *j* = 1, 2 and *k* = 1, 2, 3, 4 were for the *i*th vehicle). The subscripts “*c*”, “*t*_1_”, “*t*_2_”, and “*w*” denote the vehicle body, front bogie, rear bogie, and wheel-set, respectively. In total, the railway vehicle model developed in this study had 30 DOFs. The suspension connections between the car body and bogies and between the bogies and wheelsets were modeled by linear spring-dashpots.

The total potential energy of train inertia forces includes individual potential energy associated with inertia forces (e.g., car body, front and rear bogies, and four wheel-sets), elastic strain of linear springs, damping forces of dashpots (e.g., primary and secondary suspension systems), and the potential gravitational energy of the whole vehicle (e.g., car body, front and rear bogies, and four wheel-sets). According to the principle of conservation of the total potential energy of dynamic elastic system [25], the multi-body dynamics (MBD) based matrix expression of the governing equation of spatial train vibration (see Equation (1)) was obtained by using the “right number seating” rule [25].

(1) $$M_{v}{\ddot{X}}_{v}+C_{v}{\dot{X}}_{v}+K_{v}X_{v}=F_{v}$$

where ***M_v_***, ***C_v_***, and ***K_v_*** denote the mass, damping, and stiffness matrices of the railway vehicles, respectively; {***X_v_***} is the corresponding displacement vector; and ***F_v_*** is the force vector generated from the wheel–rail interactions including the excitation of track irregularities. More details on forming these matrices are described elsewhere [24].

Since the wheel-sets were in full contact with the rail beams, the wheel–rail interaction consisted of normal contact forces formulated by the Hertz theory of normal elastic contact [26] and tangential contact forces formulated by the Kalker linear rolling contact theory [27]. More details about wheel–rail interaction, contact forces, and wheel–rail creep force models are not included herein due to space limitation and can be found elsewhere [28,29,30]. Despite either two-dimensional (2D) or 3D half-train models prevail in the existing literature (e.g., [31]), the 3D full-train model capable of considering spatial motion and wheel/rail interaction more realistically was developed in this study. On the other hand, the ballast layer was commonly modelled and meshed by using the finite element method (FEM) in the existing literature (e.g., [31]), whereas the numerical model developed in this study treated the ballast layer as a series of mass-dashpot blocks so as to better capture and reflect the discrete, particulate nature of ballast materials. Those are the highlights of the major novelties and advantages of the numerical model developed in this study as compared to those presented in the existing literature.

### 2.3. Modeling the Ballasted Track and Underlying Subgrade

**Figure 3:**
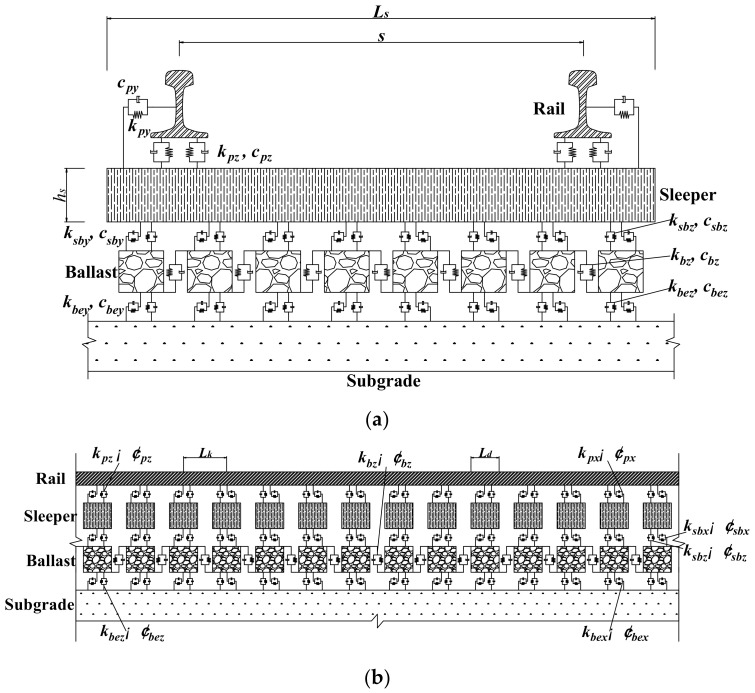
Illustration of the ballasted track model developed: (**a**) transverse section view and (**b**) longitudinal section view.

The finite element method was used to model the ballasted track substructure of the train-ballasted track-subgrade coupling system. The ballasted track was composed of rail beams, sleepers, the ballast layer, and the fastening system. The rail beams were simulated by the Euler beam elements. The sleepers were regarded as rigid bodies characterized by the combination of lateral motion *x*, longitudinal motion *y*, vertical motion *z*, roll motion *θ*, pitch motion *φ*, and yaw motion *Ψ*. The ballast layer was assumed to be mass blocks featuring 3 DOFs in *x*, *y*, and *z* directions. The interactions between rail beams and sleepers and between sleepers and the ballast layer were modeled by linear spring-dashpot elements, as shown in Figure 3.

In Figure 3, ***L_s_*** denotes the length of a sleeper, ***L_d_*** denotes the width of a sleeper, ***h_s_*** denotes the thickness of a sleeper, ***L_k_*** denotes the distance between adjacent sleepers, s denotes the distance between the center of left rail and right rail, [***k_px_***, ***k_py_***, ***k_pz_***] denote the longitudinal, lateral, and vertical stiffness of fastener, [***c_px_***, ***c_py_***, ***c_pz_***] denote the longitudinal, lateral, and vertical damping of fastener, [***k_sbx_***, ***k_sby_***, ***k_sbz_***] denote the longitudinal, lateral, and vertical stiffness between sleeper and ballast, [***c_sbx_***, ***c_sby_***, ***c_sbz_***] denote the longitudinal, lateral, and vertical damping between sleeper and ballast, [***k_bex_***, ***k_bey_***, ***k_bez_***] denote the longitudinal, lateral, and vertical stiffness between ballast and subgrade, [***c_bex_***, ***c_bey_***, ***c_bez_***] denote the longitudinal, lateral, and vertical damping between ballast and subgrade.

The foundation part of the high-speed railway track beneath the ballast layer is relatively thick and normally consists of the top embankment layer, the bottom embankment layer, and the engineered and natural subgrade soil layers according to Chinese standards. The physical and mechanical characteristics of such foundation geomaterials at different layers are different. Under the application of transient high-speed train loading, each of such foundation layers experiences relatively small deformation. It can thus be reasonably assumed that such foundation layers still remain in a linear elastic state, making the use of elastic constitutive models applicable. For brevity, such different foundation layers were collectively named as the composite “subgrade” in this study. According to the theories of elasticity and finite element method, the 3D 8-node iso-parametric elements were used, and each element node was assumed to contain 3 translational DOFs without rotational DOFs. Therefore, a 3D 8-node solid element contained 24 DOFs. The element stiffness matrix, element damping matrix, and element mass matrix of subgrade were formulated accordingly, and the displacement vector at element nodes was expressed as Equation (2):

(2) $$\{a\}={\left\{\begin{array}{llllllllll} u_{1} & v_{1} & w_{1} & u_{2} & v_{2} & w_{2} & \cdots & u_{8} & v_{8} & w_{8} \end{array}\right\}}^{T}$$

The 3D displacement (*u*, *v*, *w*) at each node of the elements can be obtained by 3D linear Lagrange interpolation of nodal displacements as follows:

(3) $$\left\{\begin{array}{l} u=N_{1}u_{1}+N_{2}u_{2}+N_{3}u_{3}+N_{4}u_{4}+N_{5}u_{5}+N_{6}u_{6}+N_{7}u_{7}+N_{8}u_{8}\\ v=N_{1}v_{1}+N_{2}v_{2}+N_{3}v_{3}+N_{4}v_{4}+N_{5}v_{5}+N_{6}v_{6}+N_{7}v_{7}+N_{8}v_{8}\\ w=N_{1}w_{1}+N_{2}w_{2}+N_{3}w_{3}+N_{4}w_{4}+N_{5}w_{5}+N_{6}w_{6}+N_{7}w_{7}+N_{8}w_{8} \end{array}\right.$$

which can be rewritten in the matrix form as shown in Equation (4):

(4) $$\left\{\begin{array}{l} u\\ v\\ w \end{array}\right\}=\left[\begin{array}{ccccccc} N_{1} & 0 & 0 & \cdots & N_{8} & 0 & 0\\ 0 & N_{1} & 0 & \cdots & 0 & N_{8} & 0\\ 0 & 0 & N_{1} & \cdots & 0 & 0 & N_{8} \end{array}\right]\left\{\begin{array}{c} u_{1}\\ v_{1}\\ w_{1}\\ \cdots \\ u_{8}\\ v_{8}\\ w_{8} \end{array}\right\}=\left[N]\{a\right\}$$

**Figure 4:**
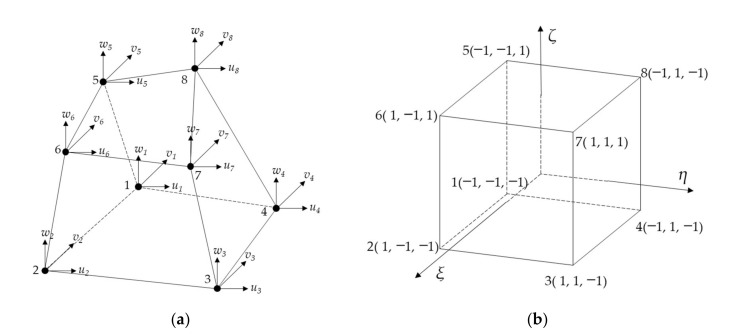
Illustration of the 3D iso-parametric element sketched in both (**a**) global coordinate system and (**b**) local coordinate system.

The schematic diagram of the 3D iso-parametric element is shown in Figure 4.

The shape functions of each element can be written as Equation (5):

(5) $$\left\{\begin{array}{l} N_{1}=1/8(1-\xi )(1-\eta )(1-\zeta )\\ N_{2}=1/8(1+\xi )(1-\eta )(1-\zeta )\\ N_{3}=1/8(1+\xi )(1+\eta )(1-\zeta )\\ N_{4}=1/8(1-\xi )(1+\eta )(1-\zeta )\\ N_{5}=1/8(1-\xi )(1-\eta )(1+\zeta )\\ N_{6}=1/8(1+\xi )(1-\eta )(1+\zeta )\\ N_{7}=1/8(1+\xi )(1+\eta )(1+\zeta )\\ N_{8}=1/8(1-\xi )(1+\eta )(1+\zeta ) \end{array}\right.$$

where ξ∈[−1,1], η∈[−1,1],ζ∈[−1,1].

The Jacobian matrix of each element can be obtained from the above shape functions as follows:

(6) $$[J]=\left[\begin{array}{lll} \frac{\partial x}{\partial \xi } & \frac{\partial y}{\partial \xi } & \frac{\partial z}{\partial \xi }\\ \frac{\partial x}{\partial \eta } & \frac{\partial y}{\partial \eta } & \frac{\partial z}{\partial \eta }\\ \frac{\partial x}{\partial \zeta } & \frac{\partial y}{\partial \zeta } & \frac{\partial z}{\partial \zeta } \end{array}\right]=\left[\begin{array}{lll} \sum_{i=1}^{8}\frac{\partial N_{i}}{\partial \xi }x_{i} & \sum_{i=1}^{8}\frac{\partial N_{i}}{\partial \xi }y_{i} & \sum_{i=1}^{8}\frac{\partial N_{i}}{\partial \xi }z_{i}\\ \sum_{i=1}^{8}\frac{\partial N_{i}}{\partial \eta }x_{i} & \sum_{i=1}^{8}\frac{\partial N_{i}}{\partial \eta }y_{i} & \sum_{i=1}^{8}\frac{\partial N_{i}}{\partial \eta }z_{i}\\ \sum_{i=1}^{8}\frac{\partial N_{i}}{\partial \zeta }x_{i} & \sum_{i=1}^{8}\frac{\partial N_{i}}{\partial \zeta }y_{i} & \sum_{i=1}^{8}\frac{\partial N_{i}}{\partial \zeta }z_{i} \end{array}\right]$$

Then the nodal strain matrix can be obtained and shown in Equation (7):

(7) $$\varepsilon_{i}=\left[\begin{array}{ccc} \partial N_{i}/\partial x & 0 & 0\\ 0 & \partial N_{i}/\partial x & 0\\ 0 & 0 & \partial N_{i}/\partial x\\ 0 & \partial N_{i}/\partial z & \partial N_{i}/\partial y\\ \partial N_{i}/\partial z & 0 & \partial N_{i}/\partial x\\ \partial N_{i}/\partial y & \partial N_{i}/\partial x & 0 \end{array}\right];\varepsilon =\left[\begin{array}{cccccccc} \varepsilon_{1} & \varepsilon_{2} & \varepsilon_{3} & \varepsilon_{4} & \varepsilon_{5} & \varepsilon_{6} & \varepsilon_{7} & \varepsilon_{8} \end{array}\right]$$

According to the constitutive equations of elasticity, the element stress matrix can be expressed as {*σ*} = [***D***]{***ε***}, where [***D***] is the symmetric elastic stiffness matrix and can be written as Equation (8):

(8) $$[D]=\frac{E(1-\mu )}{\left(1+\mu )(1-2\mu \right)}\left[\begin{array}{cccccc} 1 & \frac{\mu }{1-\mu } & \frac{\mu }{1-\mu } & 0 & 0 & 0\\ \frac{\mu }{1-\mu } & 1 & \frac{\mu }{1-\mu } & 0 & 0 & 0\\ \frac{\mu }{1-\mu } & \frac{\mu }{1-\mu } & 1 & 0 & 0 & 0\\ 0 & 0 & 0 & \frac{1-2\mu }{2(1-\mu )} & 0 & 0\\ 0 & 0 & 0 & 0 & \frac{1-2\mu }{2(1-\mu )} & 0\\ 0 & 0 & 0 & 0 & 0 & \frac{1-2\mu }{2(1-\mu )} \end{array}\right]$$

where *μ* is Poisson’s ratio and *E* is elastic modulus.

According to the principle of minimum potential energy, the stiffness matrix [***k^e^***] of a 3D 8-node iso-parametric element can be formulated as Equation (9):

(9) $$\left[K^{e}\right]={\int_{-1}^{1}\int_{-1}^{1}\int_{-1}^{1}\left[B\right]}^{T}\left[D\right]\left[B\right]\det\left(\left[J\right]\right)d\xi d\eta d\zeta$$

Then the mass matrix [*M^e^*] of each element can be obtained as Equation (10):

(10) $$\left[M^{e}\right]={\int_{-1}^{1}\int_{-1}^{1}\int_{-1}^{1}\rho \left[N\right]}^{T}\left[N\right]\det\left(\left[J\right]\right)d\xi d\eta d\zeta$$

The damping matrix of each element can be obtained by calculating Rayleigh damping as Equation (11):

(11) $$\left[C^{e}\right]=\alpha \times \left[K^{e}\right]+\beta \times \left[M^{e}\right]$$

The coupling method between different structural layers and the determination of boundary conditions are detailed elsewhere [32]. The dynamic governing equation of the 3D train-ballasted track-subgrade coupling system can be finally formulated as Equation (12):

(12) $$\left[\begin{array}{cc} M_{t} & \\ & M_{s} \end{array}\right]\left\{\begin{array}{c} {\ddot{X}}_{t}\\ {\ddot{X}}_{s} \end{array}\right\}+\left[\begin{array}{cc} C_{t} & C_{ts}\\ C_{st} & C_{s} \end{array}\right]\left\{\begin{array}{c} {\dot{X}}_{t}\\ {\dot{X}}_{s} \end{array}\right\}+\left[\begin{array}{cc} K_{t} & K_{ts}\\ K_{st} & K_{s} \end{array}\right]\left\{\begin{array}{c} X_{t}\\ X_{s} \end{array}\right\}=\left\{\begin{array}{c} F_{t}\\ F_{s} \end{array}\right\}$$

After considering the wheel–rail interaction and track irregularity, the above dynamic governing equation in Equation (12) can be rewritten as Equation (13):

(13) $$\left[\begin{array}{ccc} M_{v} & & \\ & M_{t} & \\ & & M_{s} \end{array}\right]\left\{\begin{array}{c} {\ddot{X}}_{v}\\ {\ddot{X}}_{t}\\ {\ddot{X}}_{s} \end{array}\right\}+\left[\begin{array}{ccc} C_{v} & C_{vt} & \\ C_{tv} & C_{t} & C_{ts}\\ & C_{st} & C_{s} \end{array}\right]\left\{\begin{array}{c} {\dot{X}}_{v}\\ {\dot{X}}_{t}\\ {\dot{X}}_{s} \end{array}\right\}+\left[\begin{array}{ccc} K_{v} & K_{vt} & \\ K_{tv} & K_{t} & K_{ts}\\ & K_{st} & K_{s} \end{array}\right]\left\{\begin{array}{c} X_{v}\\ X_{t}\\ X_{s} \end{array}\right\}=\left\{\begin{array}{c} F_{v}\\ F_{t}\\ F_{s} \end{array}\right\}$$

**Figure 5:**
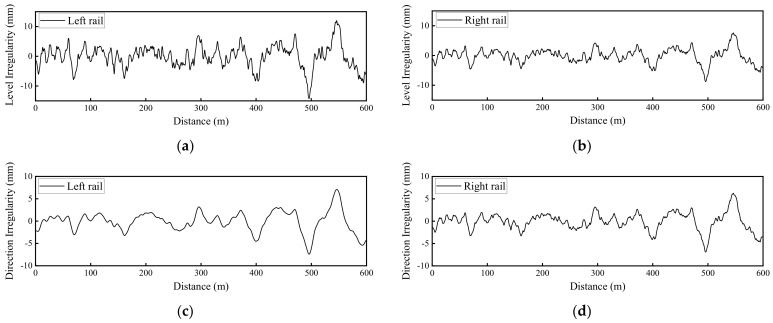
Illustration of the spectrum sample of track irregularity used: (**a**) Level irregularity of left rail; (**b**) level irregularity of right rail; (**c**) direction irregularity of left rail; and (**d**) direction irregularity of right rail.

Since the focus of this study was on the influence of rail fastener failure, only a preselected deterministic sample of track irregularity spectrum was considered, i.e., the classic German low-interference track irregularity spectrum, as shown in Figure 5. The method of obtaining this spectrum can be found elsewhere [30,33]. Note that in Figure 5, the “level irregularity” denotes the irregularity of rail surface along the track extension direction, whereas the “direction irregularity” denotes the irregularity of track centerline along the track extension direction.

## 3. Verification of the Numerical Model Established

### 3.1. Model Verification by Field Testing Data of Ballasted Track of Heavy-Haul Railway

**Figure 6:**
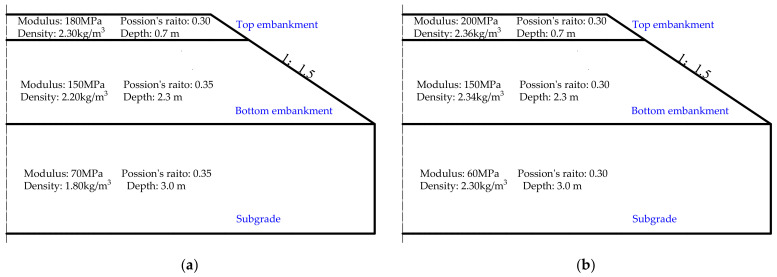
Illustration of two different subgrade configurations used for verifying the numerical model developed: (**a**) Case 1—Heavy-haul railway and (**b**) Case 2—High-speed railway.

In the literature surveyed, relatively few field-measured data are available for ballasted track of high-speed railway, while a large number of field tests have been carried out for ballastless concrete slab track of high-speed railway. The ballasted track has traditionally been adopted for heavy-haul railways due to its ability to sustain high axle load and large tonnage, and substantial field tests have been carried out on heavy-haul railways. The modeling methods of high-speed and heavy-haul trains are similar, whereas the modeling methods of ballasted tracks used in high-speed and heavy-haul railways are identical. Therefore, the FEM-based numerical model of the train-ballasted track-subgrade coupling system as established in this study was verified against the field-measured data of heavy-haul railway collected from the literature [34,35]. Specifically, the heavy-haul train with axle load of 25 tons and travel speed of 80 km/h was selected for modeling, but the train parameters were reasonably simplified on the basis of the original reference [34]. The field-measured data described elsewhere [35] were selected for model verification. The key model input parameters of foundational layers are shown in Figure 6a. The maximum vertical displacement values of rails and sleepers that were measured in the field and calculated by the numerical model are summarized in Table 1 for comparison. It can be seen from Table 1 that the differences between model-simulated and field-measured dynamic responses are quite small, which successfully verified the accuracy and validity of the numerical model established in this study.

### 3.2. Model Verification by Field Testing Data of Ballasted Track of High-Speed Railway

**Figure 7:**
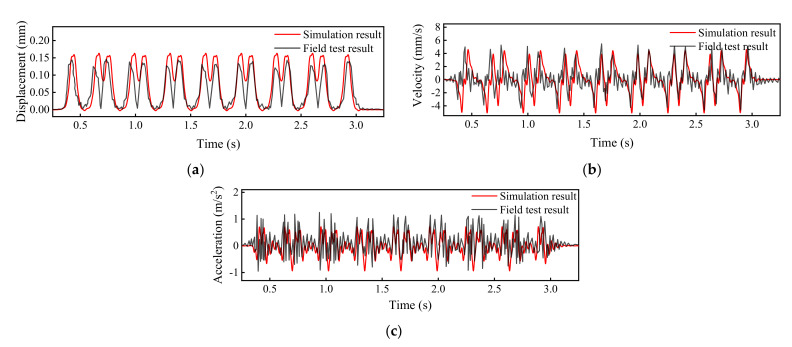
Comparisons of field-measured and model-calculated dynamic responses of the top embankment layer: (**a**) Vertical displacement; (**b**) vertical velocity; and (**c**) vertical acceleration.

The field data of ballasted track of high-speed railway measured by instrumented sensors were used in this study for model verification with more details described elsewhere [36]. The high-speed railway vehicles were of the CRH380AJ-203 type, of which the configuration can be found elsewhere [36]. The running speed of the high-speed train was 295 km/h. The key model input parameters of foundational layers are shown in Figure 6b. The comparisons of field-testing data and model simulation results are shown in Figure 7. Note that the comparison was made for about 3 s only because the time duration of the real train passage in the field was about 3 s at the train speed of 295 km/h, during which the field measurements of dynamic responses were collected. Greater time duration of the real train passage selected for model verification could permit such comparison for a longer time.

It can be seen from Figure 7 that the field-measured and model-simulated amplitudes of vertical displacement of the top embankment layer are about 0.14 mm and 0.16 mm, respectively, and that both field-measured and model-simulated amplitudes of vertical velocity and vertical acceleration of the top embankment layer are about 5 mm/s and 1 m/s^2^, respectively. The model-calculated results of all the above-mentioned dynamic responses of the top embankment layer matched well the field-measured data, which further verified the validity and accuracy of the numerical model established in this study.

## 4. Numerical Simulation Results and Analysis

**Figure 8:**
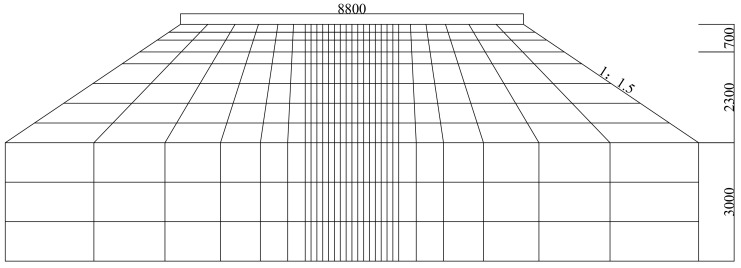
The schematic illustration (not to scale) of the composite subgrade system in the numerical model (unit: mm).

**Figure 9:**
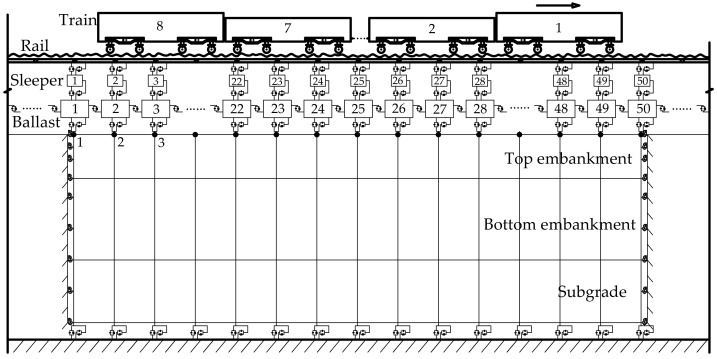
Global configuration of the 3D train-ballasted track-subgrade coupling system.

The composite “subgrade” model was established in this study according to the design drawings of foundation layers supporting single ballasted tracks of China’s high-speed railway [37]. The longitudinal length of the composite subgrade model was selected as 30 m to eliminate the boundary effects while balancing computational cost. The cross-sectional configuration of the composite subgrade is illustrated in Figure 8. The parameters of high-speed railway vehicles in the numerical model are listed in Appendix A. The global configuration of 3D train-ballasted track-subgrade system is sketched in Figure 9.

As described previously, the composite subgrade was modelled as linear elastic in the current numerical model developed, and only elastic modulus and Poisson’s ratio of the subgrade were required as mechanistic inputs in the numerical model (see Table A2 in the Appendix A). From this perspective, it can be applicable to both static and dynamic analyses. It is worth noting that such elastic modulus of the subgrade was averaged from corresponding stress-dependent resilient modulus values calculated at typical stress states in the subgrade as induced by moving train loading. Those resilient modulus values were referenced from the comprehensive laboratory testing database archived for different types of subgrade materials under varying combinations of material physical conditions and stress paths; however, the corresponding details of the testing standards, procedures, and results are definitely out of the scope and can be found elsewhere for brevity [38]. In contrast, the ballast layer was modelled as a series of mass-dashpot blocks in the numerical model; therefore, the related material properties of such ballast layer cannot be directly obtained from laboratory or field tests. Instead, such material properties of the ballast layer were cited from elsewhere [32], which is reasonably acceptable as the variation of such material properties is regarded as trivial.

### 4.1. Dynamic Track Responses without Rail Fastner Failure

**Figure 10:**
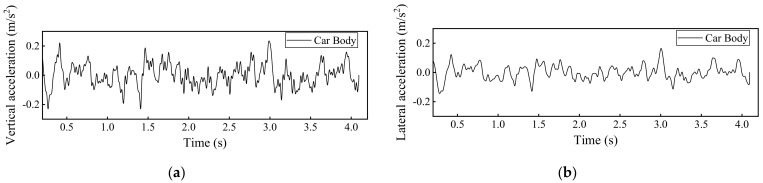
Model-calculated dynamic responses of the first passenger vehicle (the No. 2 railcar): (**a**) Vertical acceleration and (**b**) lateral acceleration.

**Figure 11:**
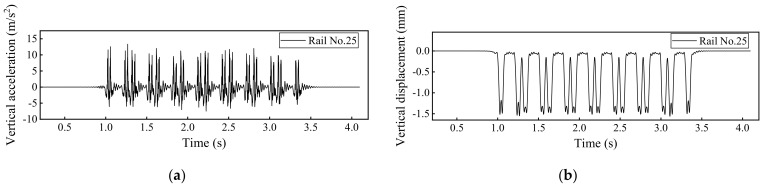
Model-calculated dynamic responses of the No. 25 rail: (**a**) Vertical acceleration and (**b**) vertical displacement.

**Figure 12:**
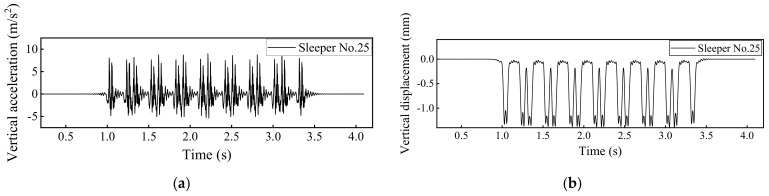
Model-calculated dynamic responses of the No. 25 sleeper: (**a**) Vertical acceleration and (**b**) vertical displacement.

**Figure 13:**
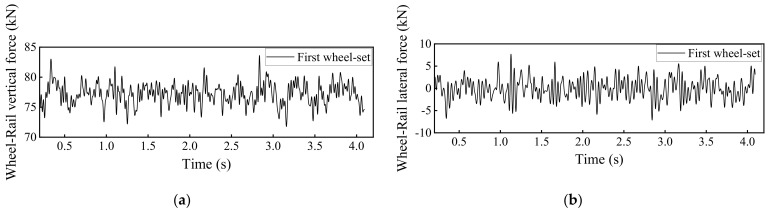
Model-calculated wheel-rail contact forces in the (**a**) vertical direction and (**b**) lateral direction.

By using the developed 3D train-ballasted track-subgrade coupling model with input parameters of the railway vehicles, rail beams, the ballast layer, and the subgrade layer listed in Appendix A, the dynamic responses of car bodies, rails, and sleepers without rail fastener failure can be calculated and shown in Figure 10, Figure 11, Figure 12 and Figure 13. Note that the term “Car body” in those figures refers to the first passenger vehicle (i.e., the No. 2 railcar shown in Figure 1) and the term “First wheel-set” refers to the first passenger vehicle’s first wheel-set (i.e., the red wheel shown in Figure 1). The running speed of railway vehicles was set as 300 km/h.

According to relevant design codes of high-speed railway [39,40], there are limits enforced on the dynamic responses of each part of the rail track system during high-speed train operation, as summarized in Table 2. Note that the parameter P_w_ in Table 2 denotes the static wheel-load of one wheel-set.

The Sperling index was used to evaluate the running stability of high-speed vehicles [40], of which the calculation equation is shown in Equation (14).

(14) $$W=7.08{\left[\frac{A^{3}}{f}F(f)\right]}^{0.1}$$

where *W* is the Sperling stability index, *A* is the acceleration of car bodies (g), *f* is the vibration frequency (Hz), and *F*(*f*) is the correction factor of frequency (see Table 3). The criteria for ranking and rating the Sperling stability index of railway vehicles are shown in Table 4 [40]. The calculated results of the Sperling stability indices without rail fastener failure are tabulated in Table 5. According to the ranking and rating criteria of the Sperling stability index in Table 5, the running stability of the high-speed railway vehicles is ranked at the first level (i.e., rated as excellent) in both vertical and lateral directions.

### 4.2. Different Scenarios of Rail Fastener Failure

**Figure 14:**
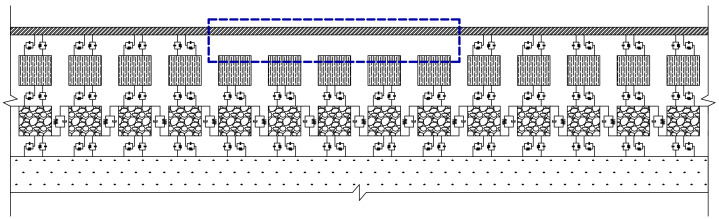
Illustration of the occurrence of one failed rail fastener (enclosed by the dashed rectangle).

When the rail fastener failure occurred, it was assumed that the linear spring-damping elements connecting the rail and the specific sleepers of interest had lost their functionality. That is, the spring stiffness and damping parameters in x, y, and z directions became zero. The example occurrence of rail fastener failure is illustrated in Figure 14.

**Figure 15:**
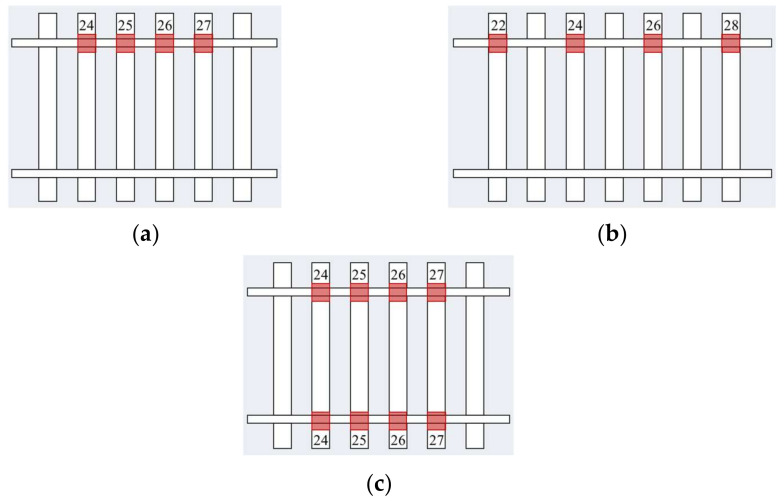
Illustration of three different patterns of failed rail fasteners: (**a**) Single-side consecutive fasteners; (**b**) single-side alternate fasteners; and (**c**) double-side consecutive fasteners.

Three different scenarios of rail fastener failure were targeted in the numerical simulations for their potential influences on dynamic track responses and running stability of high-speed railway vehicles, i.e., the failure of single-side consecutive fasteners, double-side consecutive fasteners, or single-side alternate fasteners. The calculation details of three different scenarios of rail fastener failure including the pattern, number, and location of failed rail fasteners are provided in Table 6, whereas the three different patterns of failed rail fasteners are sketched in Figure 15. In fact, the scenarios of different combinations of failed rail fasteners listed in Table 7 attempted to consider both engineering reality and potential critical (or extreme) conditions.

### 4.3. Comparison of Single-Side Fastener Failures: Consecutive versus Alternate

For brief of the analysis, the dynamic responses of car body are selected from the first passenger vehicle (No. 2), the wheel-rail force responses are selected the first wheel-set (red wheel in Figure 1) of the first passenger vehicle (No. 2).

**Figure 16:**
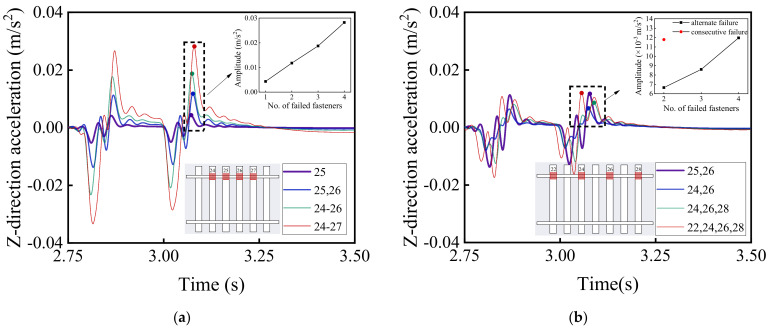
Model-calculated time history curves of vertical (Z-direction) acceleration response of car bodies with (**a**) single-side consecutive and (**b**) single-side alternate fastener failures.

**Figure 17:**
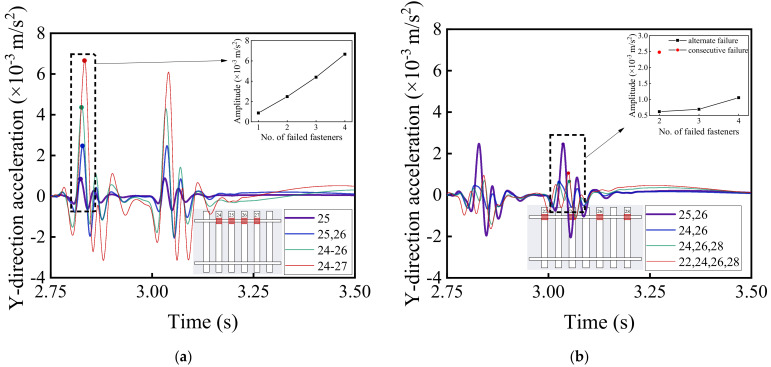
Model-calculated time history curves of lateral (Y-direction) acceleration response of car bodies with (**a**) single-side consecutive and (**b**) single-side alternate fastener failures.

Figure 16 and Figure 17 illustrate the time history curves of vertical and lateral acceleration responses of car bodies corresponding to different patterns of rail fastener failure, respectively, which reflect the running stability of high-speed railway vehicles. It can be seen from Figure 16 and Figure 17 that the rail fastener failure affects the running stability. At the locations where rail fastener failure occurs, both vertical and lateral acceleration responses of the high-speed train change suddenly. As compared with Figure 16a,b, this shows that the single-side alternate fastener failure has less impact on the running stability of high-speed railway vehicles. The variation of acceleration amplitude with the number of failed rail fasteners is also highlighted in Figure 16a,b. The influence of two single-side consecutive failed fasteners (see the red data point in Figure 16b) on the running stability of high-speed railway vehicles is similar to that of four single-side alternate failed fasteners. It is worth noting that the variation of acceleration amplitude caused by the concurrent failure of four consecutive fasteners on the same side is about three times of that caused by the concurrent failure of two consecutive fasteners on the same side. This indicates that the impact of rail fastener failure on the running stability of high-speed railway vehicles clearly exhibits a nonlinear trend.

**Figure 18:**
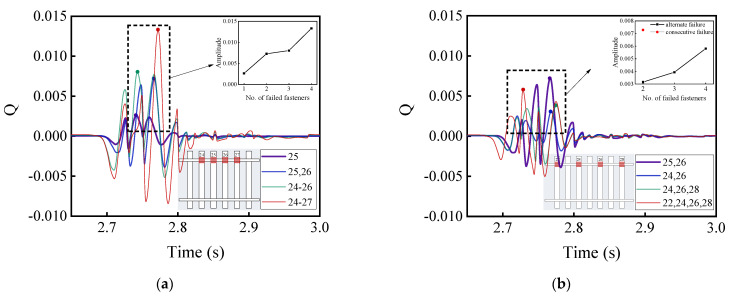
Model-calculated time-history variations of the wheel load reduction rate with (**a**) single-side consecutive and (**b**) single-side alternate fastener failures.

**Figure 19:**
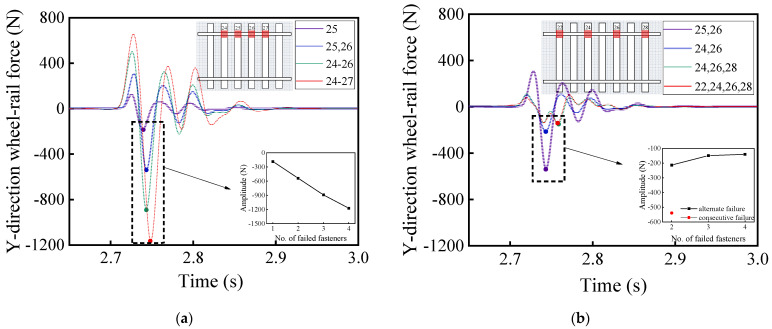
Model-calculated time-history variations of lateral wheel-rail contact force with (**a**) single-side consecutive and (**b**) single-side alternate fastener failures.

Figure 18 and Figure 19 demonstrate the time-history variations of the rate of wheel load reduction and the lateral wheel-rail contact force of the first wheel-set of passenger cars with different patterns of rail fastener failure, which reflect the operation safety of high-speed railway vehicles. As can be seen from Figure 18 and Figure 19, the different patterns of rail fastener failure would affect the driving safety, and both the wheel load reduction rate and the lateral wheel–rail contact force exhibit sudden changes at the failure locations. To be more specific, both Figure 18 and Figure 19 show that the concurrent failure of single-side alternate fasteners has less impact on train running stability than that of single-side consecutive fasteners. According to Figure 18b and Figure 19b, the concurrent failure of two single-side consecutive fasteners exerts greater impact on driving safety than that of four single-side alternate fasteners. Such calculation results clearly demonstrate and highlight the severity of consecutive fastener failure and the importance of timely identifying and properly rectifying those failures during routine railway maintenance.

**Figure 20:**
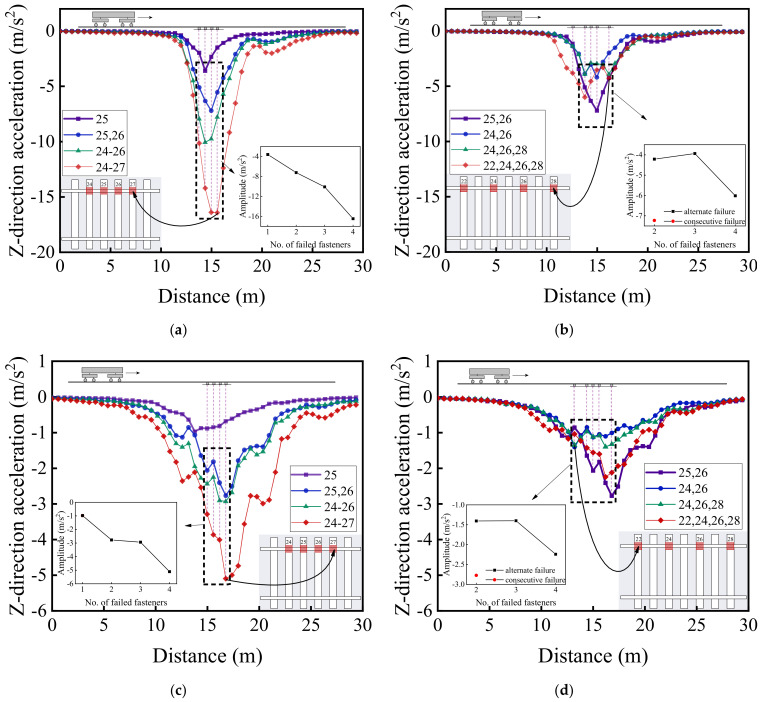
The longitudinal variations of vertical acceleration responses (from No. 1 to No. 50 rail node) of (**a**) the rail at the side with consecutively failed fasteners, (**b**) the rail at the side with alternately failed fasteners, (**c**) the rail at the opposite side of consecutively failed fasteners, and (**d**) the rail at the opposite side of alternately failed fasteners.

Figure 20 illustrates the variations of vertical acceleration responses of the rails along the vehicle running direction (from No. 1 to No. 50 rail node) on both the failure side and the opposite (intact) side for different patterns of rail fastener failure. Note that such variations represent the differences of calculated dynamic responses along the track (from No. 1 to No. 50 rail node) relative to those described in Section 4.1 corresponding to different scenarios of rail fastener failure. Regarding the impact of rail fastener failure, the trends shown in Figure 20a,b are the same as those shown in Figure 20c,d. From Figure 20a,c, it can be observed that the maximum difference of vertical rail acceleration is close to 18 m/s^2^ at the side of consecutively failed fasteners and is only about 6 m/s^2^ at the opposite side of rail fastener failure. From the enlarged sub-graphs in Figure 20b,d, the effect of two single-side, consecutively failed fasteners is similar to that of four single-side, alternately failed fasteners; further, the influence of rail fastener failure on vertical rail acceleration exhibits a nonlinear trend, as observed from the enlarged sub-graphs in Figure 20a–d.

**Figure 21:**
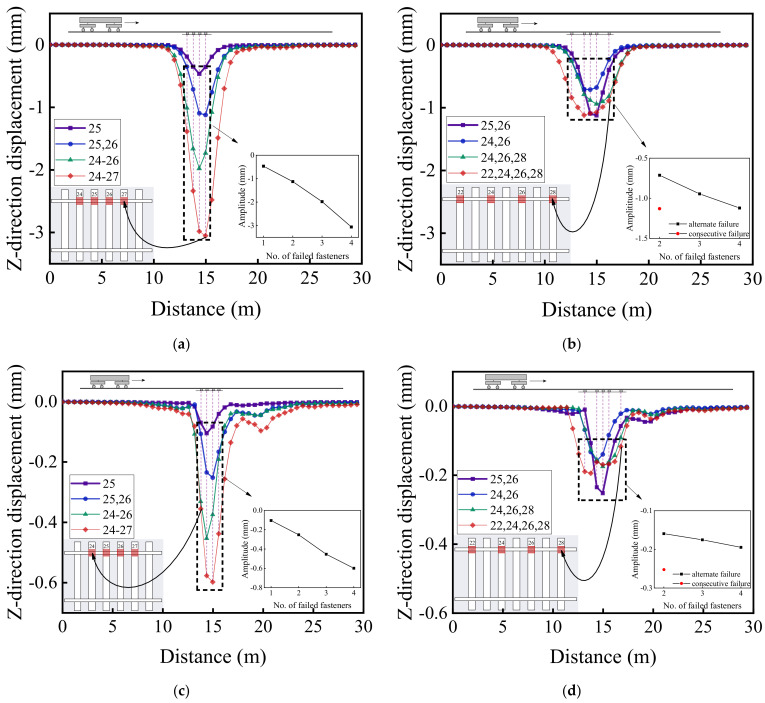
The longitudinal variations of vertical displacement responses (from No. 1 to No. 50 rail node) of (**a**) the rail at the side with consecutively failed fasteners, (**b**) the rail at the side with alternately failed fasteners, (**c**) the rail at the opposite side of consecutively failed fasteners, and (**d**) the rail at the opposite side of alternately failed fasteners.

Figure 21 illustrates the longitudinal variations of vertical displacement responses of the rails (from No. 1 to No. 50 rail node) on both the failure side and the opposite side with different patterns of rail fastener failure. From Figure 21a,c, it can be seen that the variation of vertical rail displacement at the fastener failure side is much greater than that at the opposite side, and that the maximum difference of vertical rail displacement is over 3 mm at the failed fastener side and is only about 0.6 mm at the opposite side. Similarly, the effect of single-side consecutively failed fasteners on vertical rail displacement is more significant than that of single-side alternately failed fasteners. The impact exerted by two single-side consecutively failed fasteners on the track at the fastener failure side is similar to that by four single-side alternately failed fasteners, whereas the vertical rail displacement at the opposite side of failed fasteners is more severely affected by two single-side consecutively failed fasteners. By comparing Figure 21a,b with Figure 21c,d, it can be found that the influence range of vertical rail displacement extends with increasing distance between the first and the last failed fasteners.

**Figure 22:**
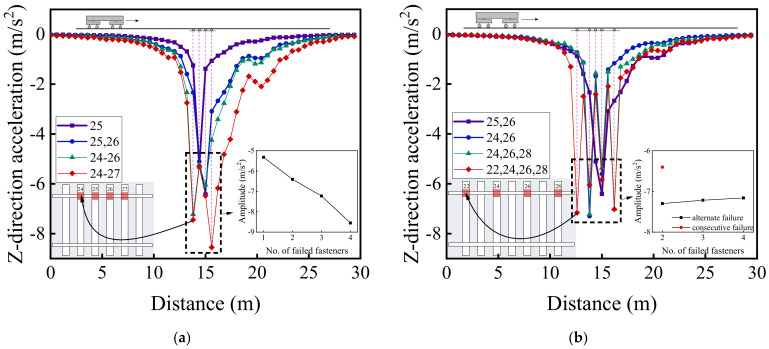
The longitudinal variations of vertical acceleration responses of sleepers (under No. 1 to No. 50 rail node) with (**a**) single-side consecutively failed fasteners and (**b**) single-side alternately failed fasteners.

**Figure 23:**
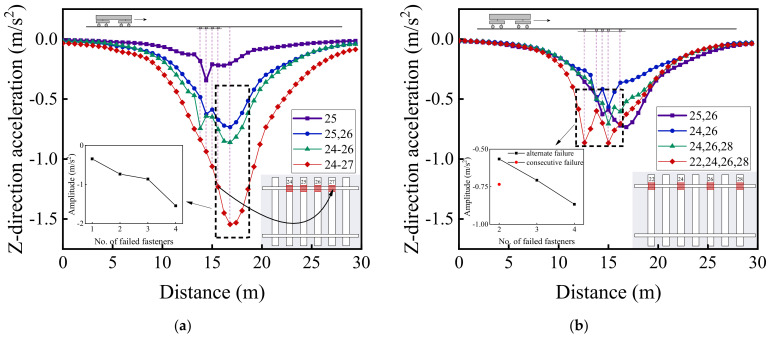
The longitudinal variations of vertical acceleration responses of ballasts (under No. 1 to No. 50 rail node) at the side with (**a**) single-side consecutively failed fasteners and (**b**) single-side alternately failed fasteners.

**Figure 24:**
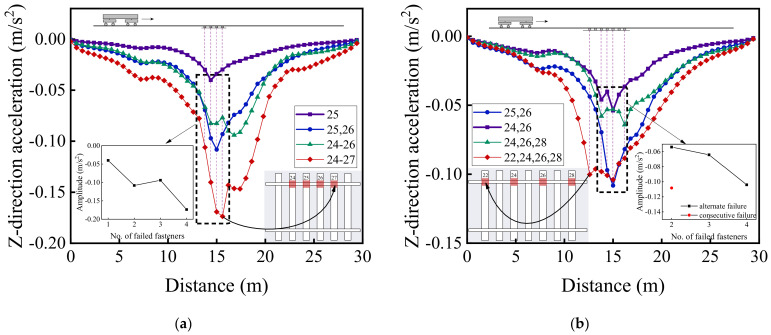
The longitudinal variations of vertical acceleration responses of subgrade surface (under No. 1 to No. 50 rail node) at the side with (**a**) single-side consecutively failed fasteners and (**b**) single-side alternately failed fasteners.

Figure 22, Figure 23 and Figure 24 illustrate the longitudinal variations of vertical acceleration responses of sleeper, ballast, and subgrade surface layer at the side with different patterns of failed fasteners. From Figure 22a, Figure 23a, and Figure 24a, it can be seen that as the number of failed fasteners increases, the acceleration responses change abruptly, the hysteresis effect of acceleration responses becomes increasingly obvious with increasing depth into the track substructure; in particular, the maximum acceleration response occurs not in the locations of failed fasteners but in the location of No. 29 fastener, as shown in Figure 23a. As for the single-side consecutive fastener failures, the impact of two consecutively failed fasteners is similar to that of three ones. When the number of failed fasteners is equal, the impact of alternately failed fasteners (e.g., No. 24 and No. 26) is less significant than that of consecutively failed ones (e.g., No. 25 and No. 26). As the depth into the track substructure increases, the impact of rail fastener failure on dynamic responses attenuates, the influence range of alternately failed fasteners extends longitudinally along the track, and the nonlinear feature of the Influencing trends becomes much more significant.

Figure 20, Figure 21, Figure 22, Figure 23 and Figure 24 show that the variation curves of acceleration responses are asymmetric and fluctuating with the influence behind the failed fasteners (relative to the train travel direction) being much more pronounced than that ahead of the failed fasteners. In contrast, the variation curves of displacement responses are approximately symmetric at the side of rail fastener failure but are asymmetric at the opposite side of rail fastener failure. This may be explained by the fact that when the single-side fastener failure occurs, the rail supporting conditions are different at both sides. Therefore, the moving vehicular loading would induce much greater dynamic responses behind the failed fasteners than ahead of the failed fasteners. Interestingly, the range of variation increases with an increasing number of failed fasteners. The alternate pattern of failed fasteners is observed to exert attenuated influence on dynamic responses of high-speed railway track structures.

### 4.4. Comparison of Rail Fastener Failures: Single-Side versus Double-Side

**Figure 25:**
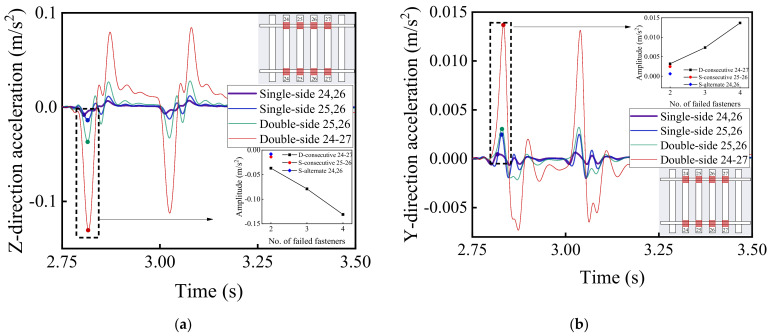
The time-history variations of (**a**) vertical and (**b**) lateral acceleration response of No. 2 railcar body (or the first passenger vehicle).

Figure 25 shows the time-history variation curves of both vertical and lateral acceleration responses of car bodies corresponding to aforementioned different patterns of rail fastener failure, i.e., two single-side alternately failed fasteners, two single-side consecutively failed fasteners, two double-side consecutively failed fasteners, and four double-side consecutively failed fasteners. It can be observed from Figure 25 that the double-side consecutive fastener failure exhibits a more severe impact on the running stability of high-speed railway vehicles. Under the circumstance of four double-side consecutively failed fasteners, the maximum difference of vertical acceleration of vehicle body reaches 0.14 m/s^2^, which is about 61% greater than its counterpart without rail fastener failure (0.23 m/s^2^). Therefore, it can be inferred that the occurrence of double-side consecutively failed fasteners would significantly compromise the ride comfort of passengers.

**Figure 26:**
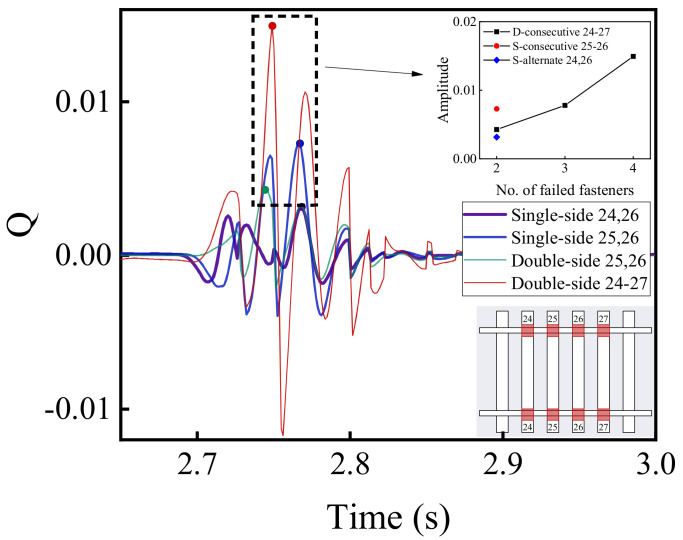
The time-history variations of wheel load reduction rate with different patterns of rail fastener failure.

**Figure 27:**
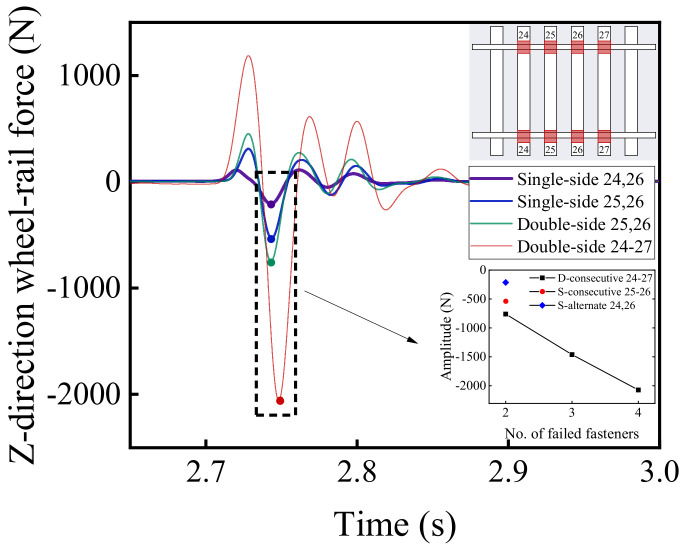
The time-history variations of lateral wheel–rail contact force with different patterns of rail fastener failure.

Figure 26 and Figure 27 demonstrate the time-history variations of the wheel load reduction rate and lateral wheel–rail contact force with the aforementioned different patterns of rail fastener failure. It can be seen from Figure 26 and Figure 27 that the double-side consecutive fastener failure is more detrimental to the running stability and safety. That is, under the circumstance of four double-side consecutive fastener failures, the maximum difference of wheel load reduction rate is about 0.015, which is approximately 100% greater than its counterpart without rail fastener failure (0.01). It thus can be inferred that the double-side consecutive fastener failure would significantly compromise the running safety of high-speed railway vehicles.

**Figure 28:**
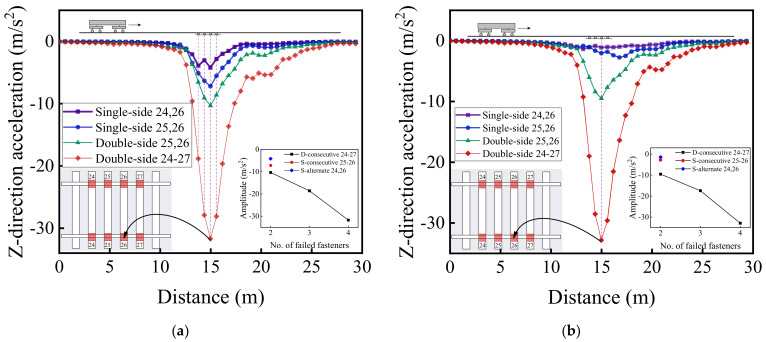

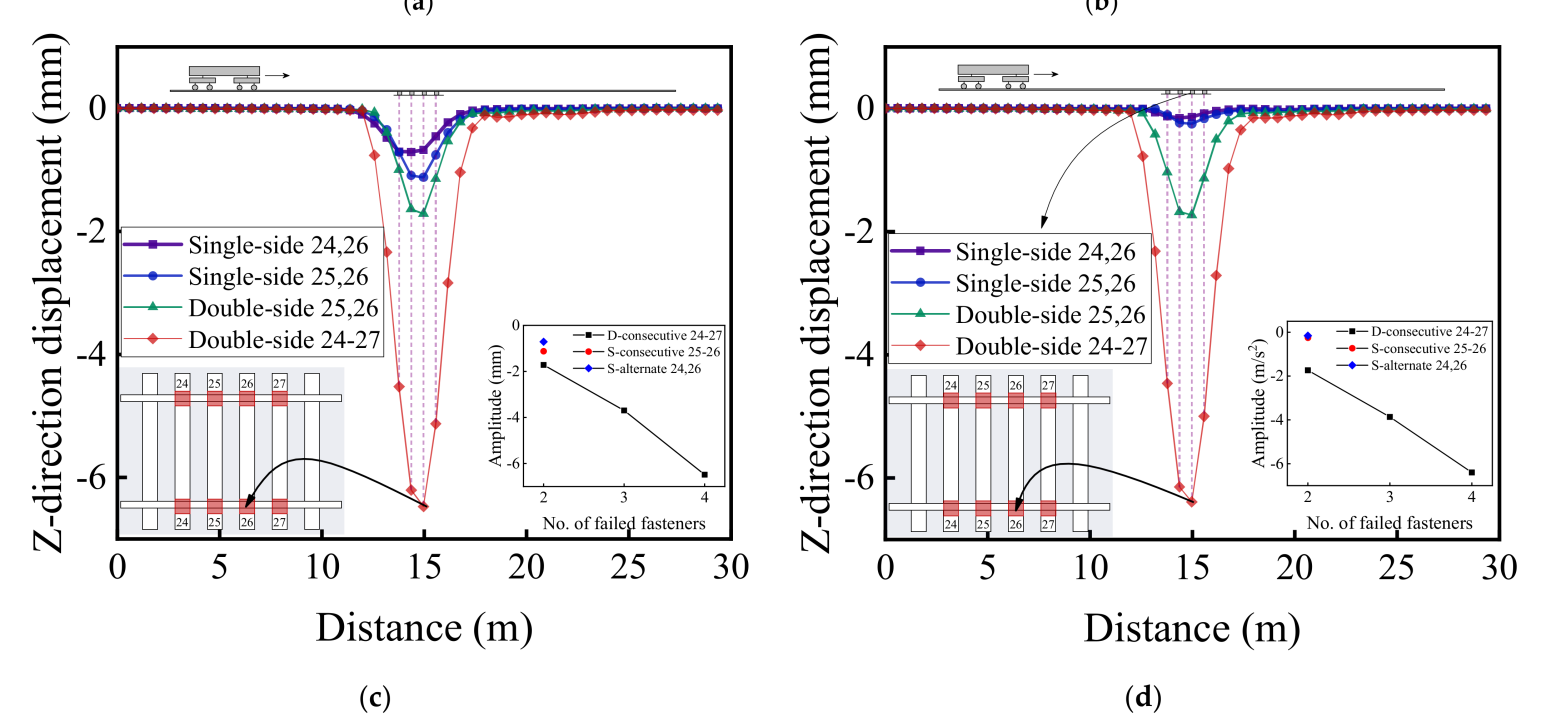
The longitudinal variations of dynamic responses of left and right rails with different patterns of rail fastener failure: (**a**) vertical acceleration of left rail; (**b**) vertical acceleration of right rail; (**c**) vertical displacement of left rail; and (**d**) vertical displacement of right rail.

Figure 28 presents the longitudinal variations of dynamic displacement and acceleration responses of both left (Figure 28a,c) and right (Figure 28b,d) rail beams. It can be seen that the acceleration and displacement responses of left and right rails are similar for double-side consecutive fastener failure, but that the effect of double-side consecutively failed fasteners is more significant than that of single-side consecutively failed fasteners. When the four consecutive fasteners fail concurrently on both sides, the maximum difference of vertical rail acceleration is roughly 35 m/s^2^ (see Figure 28a,b), and the maximum difference of vertical rail displacement is approximately 7 mm (see Figure 28c,d). Therefore, the track evenness would be significantly affected under such circumstances.

**Figure 29:**
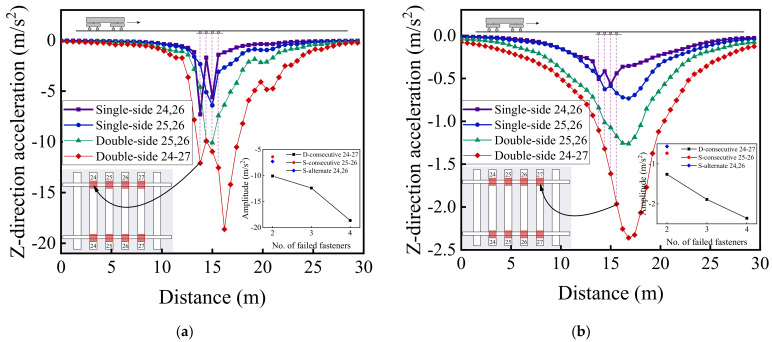
The longitudinal variations of vertical acceleration responses of (**a**) sleepers and (**b**) ballast layer under the left rail with different patterns of rail fastener failure.

**Figure 30:**
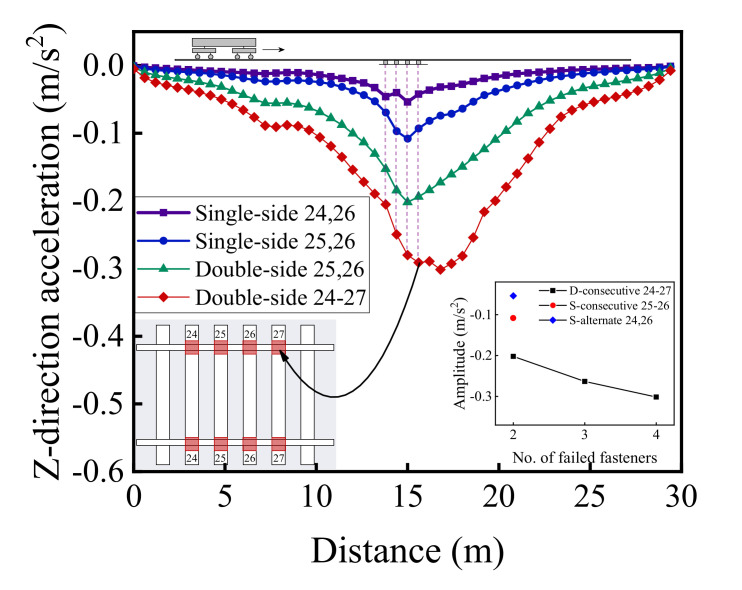
The longitudinal variation of vertical acceleration response of subgrade surface layer under the left rail with different patterns of rail fastener failure.

Figure 29 and Figure 30 illustrate the longitudinal variations of vertical acceleration of sleepers, ballast layer, and subgrade surface layer under the left rail (where single-side fastener failure occurred) ranging from No. 1 to No. 50 rail node with different patterns of rail fastener failure. It can then be seen that the influence of double-side consecutively failed fasteners on vertical acceleration responses of track substructure layers is greater than that of either single-side consecutively failed fasteners or single-side alternately failed fasteners. As the number of failed fasteners increases, the range where longitudinal dynamic responses are influenced further extends, and the influence on vertical dynamic responses attenuates with increasing depth down into track substructures. Interestingly, the hysteresis effect of acceleration responses can be clearly identified from Figure 29 and Figure 30. Among the three different patterns of rail fastener failure (i.e., double-side consecutive, single-side consecutive, and single-side alternate), the acceleration variation of each track substructure layer ahead of the failed fasteners (greater numbering) is greater than that behind the failed fasteners (smaller numbering), whereas the acceleration amplitude decreases from sleepers to underlying subgrade structure. That is, the track vibration becomes intensified after the train passes through the failed fasteners. From Figure 29 and Figure 30, we can also conclude that when double-side consecutive fastener failures occur, the track superstructure in the vicinity of failed fasteners that demands maintenance inspections spans about 15 m, i.e., 4–6 m (about 10 fasteners) backward (smaller numbering) and 9–11 m (about 20 fasteners) forward (greater numbering) from failed fasteners.

According to Table 2, Figure 11b, Figure 21 and Figure 28c,d, when the number of consecutively failed fasteners (either single-side or double-side) reaches 2 or more, the vertical displacement of rails would exceed the limit value of 2.5 mm. If the failed rail fasteners were not repaired timely, the track structures would further deteriorate. Except for the displacement response exceeding the limit value specified in related design codes, other dynamic responses are within their respective limit values specified. The calculated Sperling stability index values under different scenarios of rail fastener failure are summarized in Table 7. It can be seen from Table 7 that the rail fastener failure has an insignificant influence on the Sperling stability index, which implies that it also has little impact on the running stability of high-speed railway vehicles.

## 5. Summary and Conclusions

(1) The 3D numerical model of the train-ballasted track-subgrade coupling system was established with the integration of multibody dynamics (MBD) and finite element method (FEM) and then validated against benchmark case studies to accurately calculate dynamic responses of each structural layer of the system during the operation of high-speed railway.
(2) Among the three different patterns of rail fastener failure studied, the descending order of the severity level of their influences on dynamic responses of ballasted track structures of high-speed railway is double-side consecutive, single-side consecutive, and single-side alternate; further, the rail fastener failure affects the running stability and safety of high-speed train to a certain extent.
(3) The influence of rail fastener failure on acceleration and displacement responses of ballasted track structures exhibits notable hysteresis, which becomes increasingly significant with increasing depth into track substructure layers. When single-side fastener failure occurs, the hysteresis effect of acceleration responses is more pronounced at the opposite side than at the side of fastener failure; in contrast, when double-side fastener failure occurs, the hysteresis effect becomes even much more apparent than the former case. When double-side consecutive fastener failures occur, the track superstructure in the vicinity of failed fasteners that demands maintenance inspections spans about 15 m, i.e., 4–6 m (about 10 fasteners) backward (smaller numbering) and 9–11 m (about 20 fasteners) forward (greater numbering) from failed fasteners. Therefore, when rail fastener failure is spotted, special attention needs to be paid during routine railway maintenance to the locations behind the failed fasteners as well, especially to the ballast and subgrade layers.
(4) The rail fastener failure exerts a significant influence on dynamic responses of ballasted track structures. The variation curves of dynamic displacement responses of each structural layer near the failed fasteners are approximately symmetric, whereas the variation curves of acceleration responses are asymmetric. This indicates that the influence of rail fastener failure on the track structures behind the failed fasteners is greater than that ahead of the failed fasteners. When the number of consecutively failed fasteners exceeds 2, the vertical displacement response of ballasted track structures exceeds the limit value of 2.5 mm specified in related design codes of high-speed railway, thus necessitating timely and proper repair work in order to prevent the deterioration of rails and track.

The influences of other deteriorated or failed components of the rail fastening system such as clips and rail pads are definitely important issues for smart track condition assessment and maintenance; therefore, further research efforts are currently underway to study such issues by using the same numerical modelling scheme presented in this paper. In addition, the more advanced, realistic subgrade constitutive models (e.g., stress-dependent, moisture-sensitive, and cross-anisotropic elastoplastic ones) can be further implemented in the current numerical model established, where the more meaningful, accurate parametric study of the train-ballasted track-subgrade coupling system can be justified and performed.

## Figures and Tables

**Table 1:** Comparison of model-simulated results and field-measured data.

Dynamic Response	Model-Simulated Result	Field-Measured Data
Vertical displacement of rail/mm	2.22	2.01
Vertical displacement of sleeper/mm	1.52	1.54

**Table 2:** Limiting values of dynamic responses of high-speed rail track components.

Dynamic Response	Limit Value
Acceleration of car bodies	0.13 g (vertical direction)
	0.10 g (lateral direction)
Vertical wheel–rail contact force	170 kN
Vertical rail displacement	2.5 mm
Lateral rail displacement	2.0 mm
Rail acceleration	3000 m/s^2^
Vertical displacement of sleepers	2.0 mm
Lateral displacement of sleepers	2.0 mm
Acceleration of sleepers	500 m/s^2^
Reduction coefficient of wheel load	0.8
Lateral wheel–rail contact force	0.85 (15 + P_w_/3)

**Table 3:** The frequency correction factor for high-speed railway vehicles.

Vertical Vibration		Lateral Vibration	
0.5~5.9 Hz	*F*(*f*) = 0.325*f*^2^	0.5~5.4 Hz	*F*(*f*) = 0.8 *f*^2^
5.9~20 Hz	*F*(*f*) = 400/*f*^2^	5.4~26 Hz	*F*(*f*) = 650/*f*^2^
>20 Hz	*F*(*f*) = 1	<26 Hz	*F*(*f*) = 1

**Table 4:** The ranking criteria of the Sperling stability index W specified in China’s design code.

Rank	Level	Locomotive	Passenger
First	Excellent	<2.75	<2.5
Second	Good	2.75~3.10	2.5~2.75
Third	Qualified	3.10~3.45	2.75~3.0

**Table 5:** The calculated values of the Sperling stability index with rail fastener failure.

Direction	Vertical	Lateral
W value	1.40	1.38

**Table 6:** The calculation details of three different scenarios of rail fastener failure.

Fastener Failure Pattern	Fastener No.	Fastener Location
Single-side consecutive	1	25
	2	25, 26
	3	24, 25, 26
	4	24, 25, 26, 27
Double-side consecutive	1 × 2	25
	2 × 2	25, 26
	3 × 2	24, 25, 26
	4 × 2	24, 25, 26, 27
Single-side alternate	2	24, 26
	3	24, 26, 28
	4	22, 24, 26, 28

**Table 7:** The Sperling stability index values of high-speed railway track with single-side and double-side fastener failures.

Fastener Failure Condition	Vertical	Lateral
Single-side No. 25	1.40	1.38
Single-side No. 25–26	1.40	1.38
Single-side No. 24–26	1.40	1.38
Single-side No. 24–27	1.39	1.38
Single-side No. 24, 26	1.40	1.38
Single-side No. 24, 26, 28	1.40	1.38
Single-side No. 22, 24, 26, 28	1.39	1.38
Double-side No. 25	1.39	1.38
Double-side No. 25–26	1.39	1.38
Double-side No. 24–26	1.38	1.38
Double-side No. 24–27	1.36	1.38

## Data Availability

Not applicable.

## Appendix A

**Table A1:** Railway vehicle parameters used in the numerical model.

Parameter and Symbol	Value for Locomotives	Value for Passenger Cars	Unit
Car body mass [*m_c_*]	48,000	44,000	kg
Bogie mass [*m_t_*]	3300	2400	kg
Wheel-set mass [*m_w_*]	1780	2400	kg
Primary suspension stiffness [*k*_1*x*_, *k*_1*y*_, *k*_1*z*_]	14.68, 6.47, 1.176	15.0, 5.0, 0.7	MN/m
Primary suspension damping [*c*_1*x*_, *c*_1*y*_, *c*_1*z*_]	0.0, 0.0, 9.8	0.0, 0.0, 50	kN s/m
Secondary suspension stiffness [*k*_2*x*_, *k*_2*y*_, *k*_2*z*_]	0.167, 0.167, 0.323	0.28, 0.56, 0.30	MN/m
Secondary suspension damping [*c*_2*x*_, *c*_2*y*_, *c*_2*z*_]	67.1, 39.20, 9.8	120, 25, 60	kN s/m
Moment of inertia of car-body [*I_cx_*, *I_cy_*, *I_cz_*]	149.97, 2267.76, 2139.90	100, 2700, 2700	Mg m^2^
Moment of inertia of bogie [*I_tx_*, *I_ty_*, *I_tz_*]	2.67, 1.81, 3.30	2.4, 5.4, 5.1	Mg m^2^
Moment of inertia of wheel-set [*I_wx_*, *I_wy_*, *I_wz_*]	949, 118, 967	1200	kg m^2^
Half of bogie centroid distance [*l_c_*]	8.75	17.375/2	m
Half of wheel-set centroid distance [*l_c_*]	1.25	1.25	m
Wheel normal radius [*R_w_*]	0.43	0.43	m
Half-span of the 2nd vertical suspension system [*b*_1_]	1.0	1.0	m
Half-span of the 1st vertical suspension system [*b*_2_]	0.95	0.95	m
Half-span of the 2nd horizontal suspension system [*b*_3_]	1.0	1.0	m
Half-span of the 1st horizontal suspension system [*b*_4_]	0.95	0.95	m
Height of body above the 2nd suspension system [*h*_1_]	0.8	0.8	m
Height of the 2nd suspension system above bogie [*h*_2_]	0.3	0.2	m
Height of bogie above wheel-set [*h*_3_]	−0.05	0.1	m

**Table A2:** Model input parameters of the sub-layers in the composite subgrade.

Sub-Layer	Input Parameter	Value	Unit
Top embankment	Elastic modulus	120	MPa
	Poisson’s ratio	0.25	/
	Density	1950	kg/m^3^
	Thickness	0.7	m
Bottom embankment	Elastic modulus	110	MPa
	Poisson’s ratio	0.25	/
	Density	1800	kg/m^3^
	Thickness	2.3	m
Natural subgrade	Elastic modulus	50	MPa
	Poisson’s ratio	0.25	/
	Density	1700	kg/m^3^
	Thickness	3.0	m
